# A survey of Eocene stomach contents illuminates the origins of frugivory and seed dispersal in neornithine (crown group) birds

**DOI:** 10.1007/s00114-026-02130-2

**Published:** 2026-07-06

**Authors:** Gerald Mayr, Margaret E. Collinson

**Affiliations:** 1 Ornithological Section, Senckenberg Research Institute and Natural History Museum Frankfurt, Senckenberganlage 25, 60325 Frankfurt am Main, Germany; 2 Department of Earth Sciences, Royal Holloway University of London, Egham, Surrey TW20 OEX UK

**Keywords:** Angiosperms, Flowering plants, Frugivory, Seeds, Aves, Evolution, Messel, Ornithochory, Paleogene

## Abstract

Stomach contents with seeds in avian fossils from the latest early/earliest middle Eocene Messel oil shale (Germany) are surveyed. 20 bird specimens belonging to 10 species with seeds produced by at least 13 different plant species are reported. The fossils provide the earliest direct evidence for avian frugivory and suggest that seed dispersal by neornithine (crown group) birds occurred earlier and was more extensive than currently thought. For the first time, we report seeds in the stomach content of stem group Trogoniformes, which today are important seed dispersers in the New World tropics. Other birds belong to the Galliformes, Gruiformes (Messelornithidae), Coliiformes, Coraciiformes, and the extinct taxa Halcyornithidae and Zygodactylidae. Extant Coraciiformes are predominantly carnivorous, whereas some Galliformes and Gruiformes as well as the Coliiformes today also consume fruits. All reasonably well-preserved seeds appear to be from angiosperms, including specimens identified as Rutaceae, Mastixiaceae, and Vitaceae. Unidentified seeds represent various other plant groups. Several of the arboreal birds ingested seeds of the Vitaceae. This plant family already evolved in the Late Cretaceous, whereas arboreal Neornithes did not radiate before the early Cenozoic. As such, and at least concerning arboreal birds, these fossils provide evidence for the “recruitment hypothesis”, which suggests that early Cenozoic seed dispersers exploited an existing diversity of edible fruits.

## Introduction

Multiple extant birds consume fruits and often the seeds or fruit stones pass the digestive tract. Therefore, avian frugivory is an important factor in the reproduction of spermatophyte plants (Fell et al. 2023; Quintero et al. 2024). In extant ecosystems, dispersal by birds (ornithochory) is widespread, but even though many “bird fruits” show distinctive features – being brightly colored, scentless, and conspicuously placed (Snow 1971, 1981; Wheelwright 1988; Wheelwright and Janson 1985; Jordano 2014) –, the evolutionary history of this mutualism remains poorly known.

Several bird fossils with ingested plant disseminules (seeds or fruits) as stomach or crop contents were described from the Early Cretaceous Jehol Biota of China, but most such records stem from the very archaic taxa *Jeholornis* (Zhou and Zhang 2002; Hu et al. 2022) and *Sapeornis* (Zheng et al. 2011). Seeds are also known from the stomach content of one taxon of the Enantiornithes (*Longipteryx*, O’Connor et al. 2024), which were the dominant group of arboreal birds in the Mesozoic, and it has been argued that putative ovarian follicles in some specimens of the Confuciusornithidae and Enantiornithes likewise represent ingested plant disseminules (Mayr and Manegold 2013; Mayr et al. 2020). Extant birds (Neornithes) belong to the Ornithuromorpha, and the only Mesozoic representative of this clade known to have ingested plant disseminules is the Early Cretaceous *Eogranivora* (Zheng et al. 2018).

Initially described as being indicative of granivory, that is, “seed predation” (Zhou and Zhang 2002; Zheng et al. 2011), the stomach contents of *Jeholornis* have recently been regarded as the earliest evidence of frugivory in birds (Hu et al. 2022; O’Connor et al. 2024). This conclusion was, however, drawn from indirect evidence derived from the skull morphology of the fossil taxon and the alimentary tract of extant seed-eating birds; the identity of the disseminules swallowed by *Jeholornis* remains unknown. Based on the presumed frugivory of *Jeholornis*, it was speculated that birds already played a role as dispersers of plant disseminules in the Early Cretaceous and that they contributed to the “Cretaceous Terrestrial Revolution”, i.e., the early diversification of angiosperm plants (Hu et al. 2022; Benton et al. 2022). However, all reasonably well-preserved disseminules found in the crop or stomach contents of Early Cretaceous birds are likely to stem from gymnosperms (Zheng et al. 2011). The assumption that Early Cretaceous birds were targeting disseminules of both angiosperms and gymnosperms (Hu et al. 2022; Benton et al. 2022) is speculative and conflicts with the herbaceous and shrubby nature of Early Cretaceous angiosperm plants (Friis et al. 2011), which are unlikely to have produced disseminules the size of those ingested by *Jeholornis*.

The origin of frugivory in neornithine birds, which – at least in extant tropical and subtropical ecosystems – mainly feed on angiosperm fruits, remains elusive. With one notable exception from the late Oligocene of Germany (Mayr 2013), all Paleogene bird fossils with seeds preserved in the gastrointestinal tract stem from the latest early/earliest middle Eocene of Messel in Germany (47–48 million years ago). The paleoenvironment of this site was a paratropical forested ecosystem (Collinson et al. 2012) and more than 70 avian species have been reported from Messel, which belong to 39 family-level taxa (Mayr 2017a). Even though anecdotal previous accounts exist for ingested seeds in Messel birds, no comprehensive survey has as yet been performed, and the diversity of the ingested seeds remains unexplored. Here, we review the known bird fossils from Messel, which contain hard parts of fruits or seeds in their gastrointestinal tract, and discuss their implications for the evolution of ornithochory and frugivory.

## Materials and methods

This study focuses on birds with entire, macroscopically visible, seeds in their gastrointestinal tract. Only one of the specimens exhibits a thick layer of durable tissue such as would be present in an endocarp (fruit stone) and none are unequivocally referable to any of the endocarps described in Collinson et al. (2012). Identification of the seeds is based on comparisons with isolated seeds from Messel (Collinson et al. 2012), and in most cases it is guided by overall resemblances, that is, external macroscopic characteristics, of the exposed parts. SEM images are from samples of two seeds that were taken before preparation of the bird specimens, which are transferred in synthetic resin. Such samples were not available for other specimens. Macroscopic study of the seeds by MEC was restricted to specimens in SMF.

The fossils are in the collection of the Senckenberg Research Institute Frankfurt, Germany (SMF), Hessisches Landesmuseum Darmstadt, Germany (HLMD), Staatliches Museum für Naturkunde Karlsruhe, Germany (SMNK), and Wyoming Dinosaur Center, Thermopolis, USA (WDC).

## Results

Table 1 gives an overview of all currently known birds from Messel with seeds as stomach contents. Concerning both the birds and the seeds, there exists a fair taxonomic diversity and the 20 bird specimens with ingested seeds belong to ten species in seven higher-level taxa (Galliformes, Gruiformes, Coliiformes, Trogoniformes, Coraciiformes, Halcyornithidae, Zygodactylidae).

**Table 1:** Overview of avian specimens with ingested seeds; if not indicated otherwise, the seeds represent stomach contents, measurements in parentheses (length × width) are in mm

Bird species	Collection number	Number, size (mm) and characteristics of the seeds, as well as comments and observations	Seed identity
Galliformes			
* Paraortygoides messelensis*	SMF-ME 11599	several subcircular seeds (2.2 × 2.1), or fragments thereof, and other organic matter, associated with one large gastrolith	indeterminate
Gruiformes			
* Messelornis cristata*	SMF-ME 10985	two seeds (4.0 × 2.8); Figs. 2a and 3a	*Rutaspermum messelense* (Rutaceae)
* M. cristata*	SMF-ME 11402	single seed (4.5 × 2.5), associated with numerous grit particles	indeterminate
* M. cristata*	SMF-ME 530	single endocarp with resin bodies (7.0 × 5.5); Figs. 2b and 3b	Mastixiaceae, gen. et sp. indet.
* M. cristata*	SMF-ME 807	several elliptical seeds (3.6 × 2.6); Fig. 2h	indeterminate
* M. cristata*	SMF-ME 1181	ca. 4–6 differently-sized seeds (⁓6.4 × 4.4; 4.1 × 2.6); Fig. 2g	indeterminate
* M. cristata*	SMF-ME 912	single seed (4.8 × 4.3)	indeterminate
Coliiformes			
* Selmes absurdipes*	SMF-ME 2375	> 25 densely packed seeds (3.5 × 2.9); Figs. 1b and 2d	indeterminate
* Masillacolius brevidactylus*	SMF-ME 11322	single thick-walled, large seed (6.7 × 4.4)	indeterminate seed next to foot and close to trunk; it is uncertain whether this represents an accidental association or whether the seed stems from the gastrointestinal tract
* Eoglaucidium pallas*	SMF-ME 10795	numerous densely packed seeds (6.0 × 4.9); Fig. 1c	indeterminate
* E. pallas*	SMNK-Me 553	at least four large seeds (11.5 × 8.0); Fig. 2c	this seed was likened to the Annonaceae by Mayr and Peters (1998), but this tentative identification cannot be upheld
Trogoniformes			
* Masillatrogon pumilio*	WDC PBP-MES-6246	seven seeds (5.0 × 3.2, 5.5 × 3.8); Figs. 1a and 2j	Vitaceae, cf. *Parthenocissus britannica*
* M. pumilio*	SMF-ME 11937	> 10 small seeds (3.0 × 2.0); Figs. 1e, 2n and 3f and g	*Carpolithus* species 57
Coraciiformes			
* Primobucco frugilegus*	SMF-ME 3507	> 6 subcircular seeds (7.0 × 6.5); Fig. 2m	indeterminate
* P. frugilegus*	SMF-ME 3794	> 9 seeds (6.3 × 5.1); Figs. 1f and 2f	indeterminate
* Eocoracias brachyptera*	HLMD-Me 10474	single large seed (11.3 × 5.5)	indeterminate
Halcyornithidae			
cf. *Scopsoides feisti*	SMF-ME 2865	> 20 very small seeds (1.6 × 1.1), as well as other organic matter; Figs. 2i and 3e	cf. *Carpolithus* specie*s* 56
Zygodactylidae			
* Primozygodactylus major*	SMF-ME 1758	> 30 densely packed and smooth-walled seeds in esophagus and stomach (4.5 × 3.5); Figs. 1d and 2k	Vitaceae, cf. *Vitis* sp.
* P. major*	SMF-ME 799	several different seeds including one with ornamented seed coat (3.5 × 2.0); Figs. 2e and 3d	the larger seed with ornamented seed coat resembles *Cayratia jungii* (Vitaceae)
* Primozygodactylus* cf. *major*	SMF-ME 11938	single seed (4.3 × 2.6); Figs. 2l and 3c	Vitaceae, cf. *Vitis* sp.; this seed is identical to those ingested by SMF-ME 1758

**Fig. 1:**
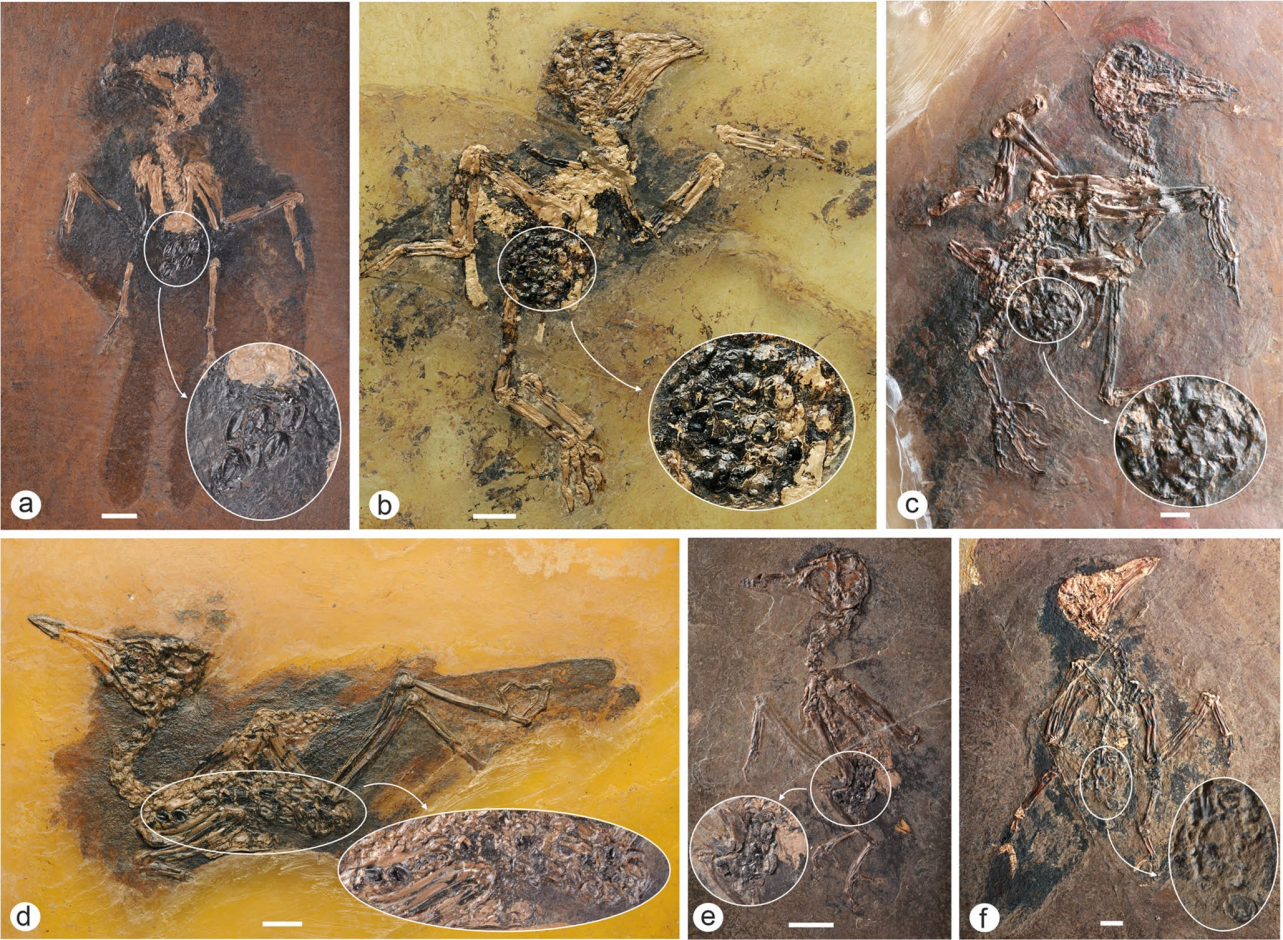
Examples of bird fossils from Messel with larger amounts of seeds in their stomach contents. **a ***Masillatrogon pumilio* (Trogoniformes; WDC PBP-MES-6246). **b ***Selmes absurdipes* (Coliiformes; SMF-ME 2375). **c ***Eoglaucidium pallas* (Coliiformes; SMF-ME 10795). **d ***Primozygodactylus major* (Parapasseres, Zygodactylidae; SMF-ME 1758 A); in this specimen, seeds are also preserved in the esophagus. **e ***M. pumilio* (Trogoniformes; SMF-ME 11937). **f ***Primobucco frugilegus* (Coraciiformes, Primobucconidae; SMF-ME 3794). The arrows denote enlarged details of the stomach contents. The scale bars equal 10 mm. Refer to Fig. 2; Table 1 for the seed details

Undetermined seeds in the stomach contents of the Messelornithidae, Coliiformes, Coraciiformes, and Zygodactylidae were mentioned before (Hesse 1990; Mayr 1998, 2018; Mayr and Peters 1998; Peters 1999; Mayr and Mourer-Chauviré 2000; Mayr et al. 2004). For three further avian clades, ingested seeds are here reported for the first time. Specimen SMF-ME 2865 is assigned to the recently described taxon *Scopsoides* (Mayr 2026) and illuminates the previously unknown diet of the Halcyornithidae. A specimen of *Paraortygoides messelensis* (SMF-ME 11599), provides the first record of ingested seeds for stem group Galliformes; in this specimen, a large gastrolith is also preserved. WDC PBP-MES-6246 and SMF-ME 11937, which are identified as *Masillatrogon pumilio*, constitute the first records of ingested seeds for representatives of the Trogoniformes (Fig. 1a).

In some specimens only one or a few seeds are present, others preserve numerous, densely packed seeds, such as the coliiform species *Eoglaucidium pallas* (SMF-ME 10795; Fig. 1c) and *Selmes absurdipes* (SMF-ME 2375; Fig. 1b) and the zygodactylid *Primozygodactylus major* (Fig. 1d; SMF-ME 1758). In some cases, more than one kind of seed is preserved (SMF-ME 1181 and SMF-ME 799), but most often there is only a single type of seed in a particular specimen.

Seed sizes range from 1.6 × 1.1 mm (SMF-ME 2865) to 11.5 × 8.0 mm (HLMD-Me 553). Specimens of the three largest bird species, that is, *Messelornis cristata*, *Eoglaucidium pallas*, and *Eocoracias brachyptera*, are also those that feature the largest seeds. With the possible exception of those preserved in *Paraortygoides messelensis*, the majority of the seeds are intact.

The disparate seed sizes and shapes indicate that at least 13 plant species are involved. Most seeds have macroscopically featureless surfaces and are therefore difficult to determine, but some exhibit distinctive structures that allow an identification.

**Fig. 2:**
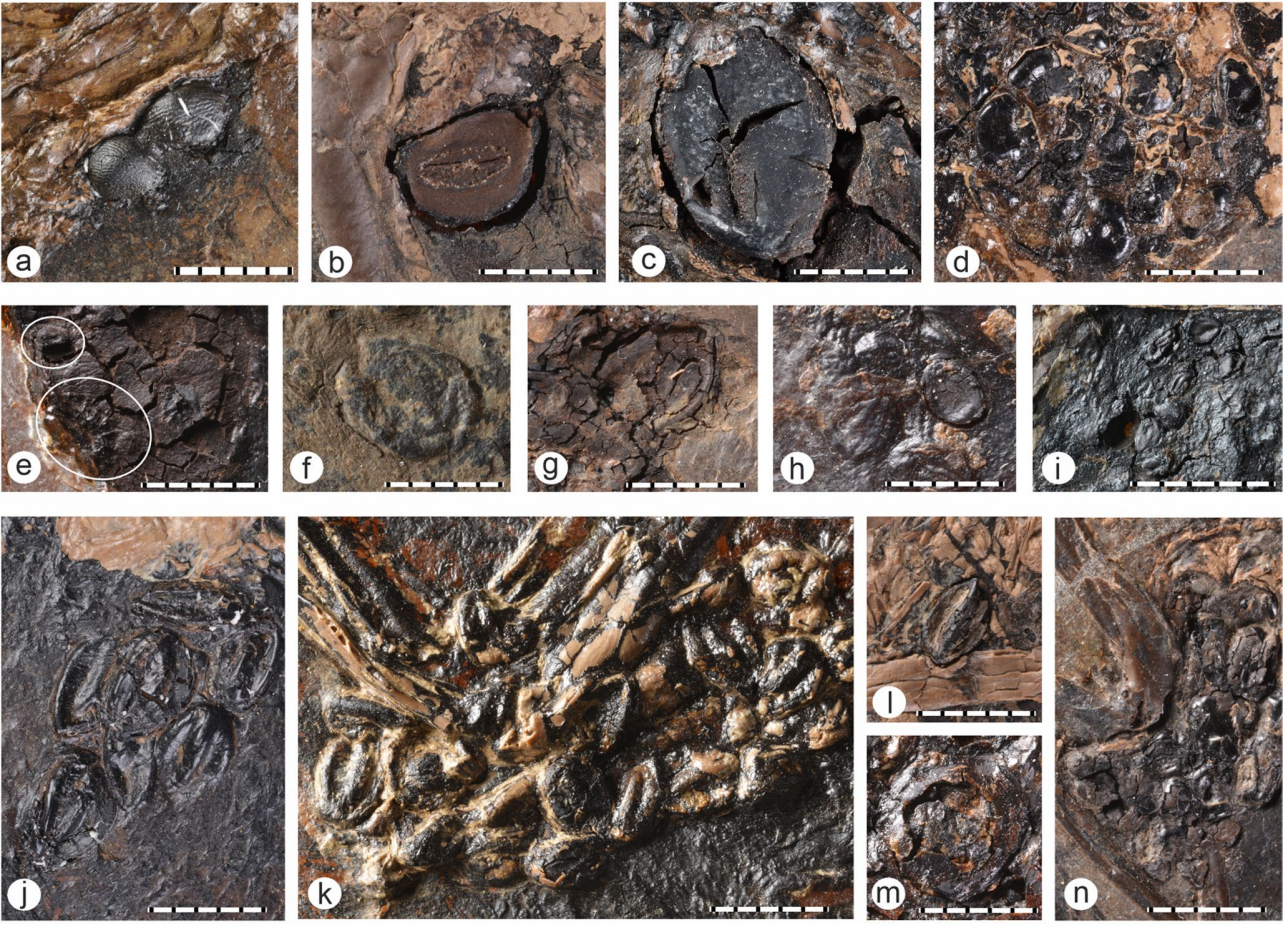
Comparison of the seeds in the stomach contents of Messel birds to show their disparity in morphology and size. **a** Two seeds of *Rutaspermum messelense* (Rutaceae) from *Messelornis cristata* (Messelornithidae; SMF-ME 10985 A). **b** Endocarp of Mastixiaceae indet. from *Messelornis cristata* (SMF-ME 530). **c** A large, undetermined seed ingested by *Eoglaucidium pallas* (Coliiformes; HLMD-Me 10474). **d** Cluster of undetermined seeds associated with *Selmes absurdipes* (Coliiformes; SMF-ME 2375); the “shininess” of these seed is due to a coating with varnish. **e** Seeds ingested by *Primozygodactylus major* (Zygodactylidae; SMF-ME 799); the circles indicate two types of differently-sized seeds, the larger one of which resembles *Cayratia jungii* (Vitaceae). **f** One of the undetermined seeds from the stomach content of *Primobucco frugilegus* (Primobucconidae; SMF-ME 3794). **g** An undetermined seed from the stomach content of *Messelornis cristata* (SMF-ME 1181). **h** Undetermined seeds associated with *M. cristata* (SMF-ME 807 A). **i** Very small seeds that resemble *Carpolithus* species 56 sensu Collinson et al. (2012) and are associated with cf. *Scopsoides feisti* (Halcyornithidae; SMF-ME 2865). **j** Seeds of the Vitaceae (cf. *Parthenocissus britannica*) associated with *Masillatrogon pumilio* (Trogoniformes; WDC PBP-MES-6246). **k** Seeds of the Vitaceae (cf. *Vitis* sp.) associated with *Primozygodactylus major* (SMF-ME 1758B). **l** Single seed of the Vitaceae (cf. *Vitis* sp.) associated with *Primozygodactylus* cf. *major* (SMF-ME 11938 A). **m** One of the unidentified subcircular seeds in the stomach contents of *Primobucco frugilegus* (SMF-ME 3507B). **n** Multiple seeds of *Carpolithus* species 57 sensu Collinson et al. (2012) associated with *Masillatrogon pumilio* (SMF-ME 11937); the “shininess” of these seeds is a real feature that characterizes this type of seed. All seeds are shown to the same size, the scale bars are in mm. Refer to Table 1 for the seed details

**Fig. 3:**
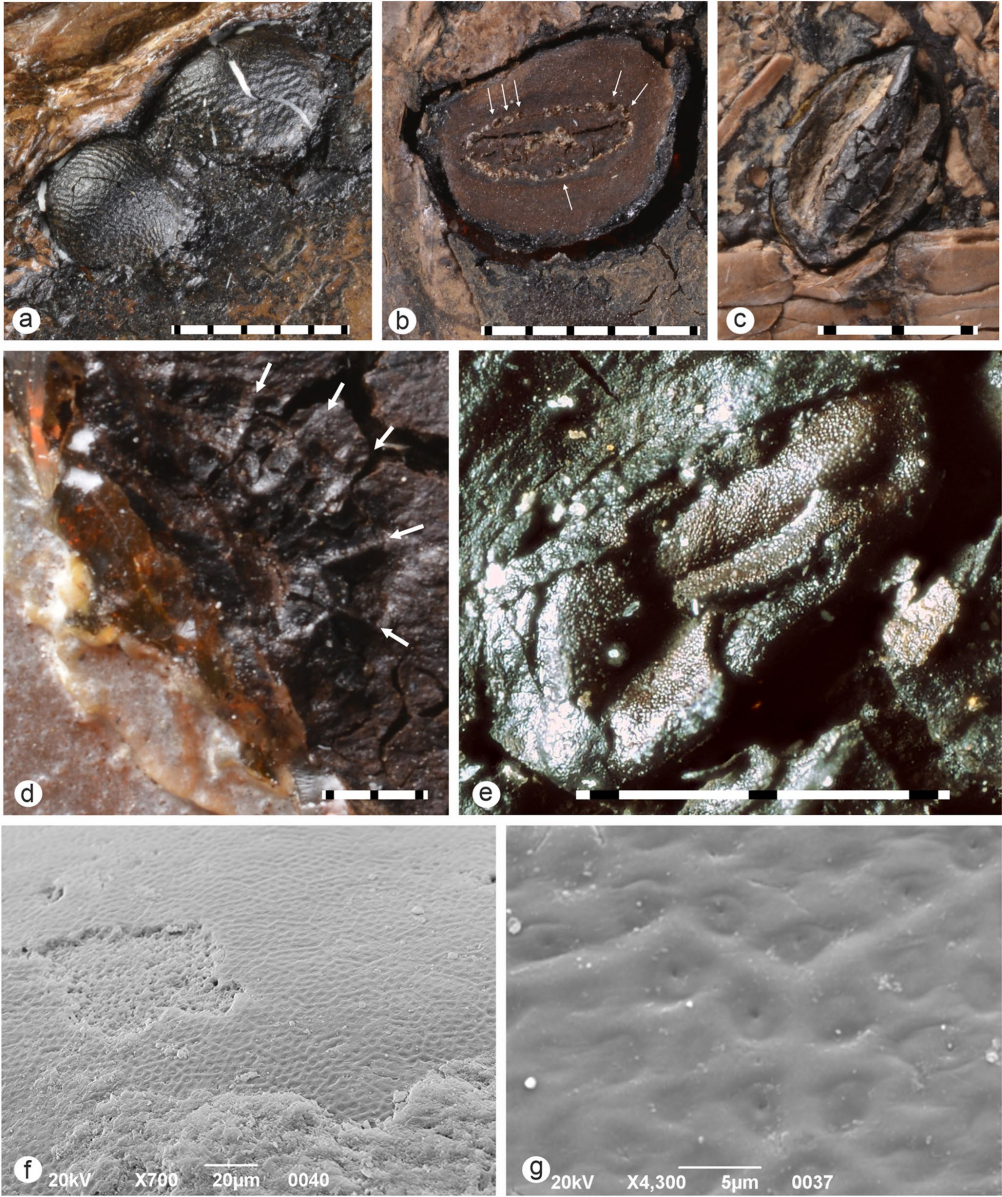
Details of selected seeds from the stomach contents of Messel birds. **a** Two seeds of *Rutaspermum messelense* (Rutaceae) from *Messelornis cristata* (SMF-ME 10985 A). **b** Endocarp of cf. Mastixiaceae indet. from *Messelornis cristata* (SMF-ME 530); the arrows point to some of the resin bodies, which are characteristic of the Mastixiaceae. **c** Seed with two longitudinal grooves interpreted as Vitaceae (cf. *Vitis* sp.) associated with *Primozygodactylus* cf. *major* (SMF-ME 11938 A). **d** A partial seed resembling *Cayratia jungii* (Vitaceae) ingested by *Primozygodactylus major* (SMF-ME 799); the arrows denote some of the radiating ridges characterizing *C. jungii*. **e** Two seeds (cf. *Carpolithus* species 56) associated with *Masillatrogon pumilio* (SMF-ME 11937); note the tiny cell outlines on the outer seed surface. **f**, **g** SEM images of the seed surface of *Carpolithus* species 57 sensu Collinson et al. (2012) associated with *M. pumilio* (SMF-ME 11937). The scale bars in **a**–**e** are in mm

Two well preserved seeds with an ornamented surface in a specimen of *Messelornis cristata* (SMF-ME 10985 A) belong to *Rutaspermum messelense* (Rutaceae; Figs. 2a and 3a), of which numerous isolated seeds are known from Messel (Collinson et al. 2012). Items ingested by a messelornithid are likely to have been derived from the Mastixiaceae, based on the presence of distinct resin bodies (Figs. 2b and 3b), which are not recorded in any other fruit or seed taxa from Messel (Collinson et al. 2012).

Seeds ingested by a specimen of the trogoniform *Masillatrogon pumilio* (SMF-ME 11937; Fig. 1e) belong to *Carpolithus* species 57 sensu Collinson et al. (2012), which has a shiny and brittle seed coat and a very characteristic surface that includes an hexagonal pattern seen in the SEM images (Fig. 3f, g). This seed type also occurs in the stomach content of one specimen of the rodent *Masillamys* from Messel (Collinson 1988). The identity of the plant that produced *Carpolithus* species 57 is unknown.

The most common seed types are interpreted as belonging to the Vitaceae (grapes), the seeds of which are characterized on the ventral side by narrow infolds and on the other, dorsal, side by a prominent, rounded or oval chalaza (Chen and Manchester 2011). In the bird specimens only the ventral seed surface is visible. Two specimens of *Primozygodactylus major* (SMF-ME 1758, SMF-ME 11938) exhibit the same type of seeds; with a size of about 4.5 × 3.5 mm, these seeds are smaller and narrower than other grape seeds from Messel. Somewhat larger seeds with broader and longer infolds are found in a specimen of *Masillatrogon pumilio* (WDC PBP-MES-6246) and agree with the seeds of *Parthenocissus britannica* in size and morphology of the exposed parts (Collinson et al. 2012). Another specimen of *P. major* (SMF-ME 799) is associated with a seed with an ornamented surface that shows radiating ridges (Fig. 3d); in size and morphology it corresponds to the seeds of *Cayratia jungii* (Vitaceae; Collinson et al. 2012); SMF-ME 799 also preserves another, much smaller seed (Fig. 2e).

The stomach contents of the halcyornithid cf. *Scopsoides feisti* (SMF-ME 11599) include very small seeds with a size of only 1.6 × 1.1 mm. These resemble *Carpolithus* species 56 of Collinson et al. (2012) and also show “isodiametric polygonal cell outlines”, which are characteristic for this seed type (Collinson et al. 2012: 238). However very small seeds are probably underrepresented in the collections from Messel because of the difficulty of recognition in the field. This limits comparisons and impedes a definitive identification. The identity of the plant that produced *Carpolithus* species 56 is unknown.

The seeds associated with other specimens (Table 1; Fig. 2) are undetermined. Judging from their sizes and shapes, they belong to at least at least five different plants, all of which are distinct from the above-mentioned ones.

## Discussion

### Taxonomic diversity of the birds and plants

Most of the seeds in the gut contents are intact, which shows that the birds were targeting other parts of the disseminules, such as fleshy parts (e.g. mesocarp), and not the seeds themselves. Therefore, the species listed in Table 1, appear to have been gulpers that swallowed entire drupes or berries.

Previous authors hypothesized that there were low levels of avian dispersers in the Eocene (Tiffney 1984; Friis et al. 2011) and that “[f]lying frugivores, birds and bats, evolved (…) mainly during the Oligocene and Miocene” (Eriksson 2016: 168). The Messel fossils show that a mutualism between neornithine birds and angiosperm seeds already existed by the latest early/earliest middle Eocene. In fact, the bird fossils from Messel with stomach contents containing seeds document a considerable diversity of both the fauna and the flora involved.

In all but one of the specimens, the seeds are stomach contents and, unlike in Early Cretaceous avians from the Chinese Jehol Formation, crop contents containing seeds are unknown from Messel. In one specimen of *Primozygodactylus major*, SMF-ME 1758, seeds are also preserved in the area of the esophagus (a distensible esophagus allows birds to store more fruits than can be digested at one time; Levey and Duke 1992). In extant birds, large crops are mainly found in granivorous species, whereas they are generally absent in frugivores (Jordano 2014).

The gruiform *Messelornis cristata* is by far the most common bird in Messel and is known from hundreds of specimens (Mayr 2017a). However, only a few individuals of this ground-dwelling species preserve seeds as stomach contents. These seeds belong to different plant species and are not found in great aggregations. Our data indicate that *M. cristata* only occasionally swallowed fruits or seeds, which may not have been readily available for these birds which would have been feeding at ground level.

Apart from the Messelornithidae, stomach contents containing seeds are mainly known from four groups of arboreal birds from Messel: the Coliiformes (Fig. 1b, c), Trogoniformes (Fig. 1a, e), Coraciiformes (Fig. 1f), and Zygodactylidae (Fig. 1d), with the latter being stem group representatives of the Passeriformes (Mayr 2022). The members of these groups belong to the Telluraves, the clade that includes most extant arboreal birds.

Today, frugivory is found in many distantly related birds, including the Tinamidae and Casuariidae, the galliform Cracidae, the gruiform Psophiidae, as well as species of the Columbiformes, Musophagiformes, Steatornithiformes, Psittaciformes, Passeriformes, Bucerotiformes, Trogoniformes, and the piciform Ramphastidae (Kissling et al. 2009). Of these extant groups, most of which occur in tropical or subtropical regions, only the Trogoniformes and Coliiformes are known from Messel. The Halcyornithidae and Zygodactylidae are representatives of the Psittacopasseres, the clade including the Psittaciformes and Passeriformes (Mayr 2022), but were ecomorphologically distinct from their extant relatives.

Undetermined ingested seeds were already reported for coliiforms from Messel (Mayr and Peters 1998; Peters 1999; Mayr 2018). It is notable that some of the species exhibit distinctive foot morphologies, with unusually short toes and a forward-directing hindtoe (Mayr and Peters 1998; Peters 1999). Possibly, these anatomical traits are related to a particular feeding behavior, in which the birds clung to some objects. However, because the seeds ingested by the coliiforms from Messel could not be determined, any correlations of this foot anatomy with a particular feeding strategy would be conjectural.

Seeds in the stomach contents of stem group representatives of the Trogoniformes are here documented for the first time and show that frugivory in trogons has also existed for at least 47 million years. However, we note that recent classifications of fossil trogoniforms as frugivores (Naware and Benson 2024) were speculative and not based on factual evidence. In fact, most extant Trogoniformes, especially those in the Old World are predominantly insectivorous, whereas frugivory is mainly found in the New World species (Collar 2001).

Several of the birds from Messel ingested large amounts of fruits (e.g., SMF-ME 2375; SMF-ME 1758) that are, therefore, likely to have been attractive for birds and probably exhibited traits found in extant bird-dispersed fruits, which are often conspicuously black or red (Wheelwright 1988). However, because fruit-eating birds show very different beak morphologies and are not characterized by unambiguous morphological traits, it cannot be determined whether any of the bird species from Messel was an obligate frugivore. These birds may well have fed on a mixed diet, which included seasonally available fruits as well as insects. 

### Evolutionary significance 

In extant ecosystems, bird-plant interactions often tend to be not very specific, even though some exceptions exist (Wheelwright 1988; Carlo et al. 2022; Lafita et al. 2026). The seed type (*Carpolithus* species 57) ingested by one individual of the trogoniform *Masillatrogon pumilio* is also found in a rodent from Messel (Collinson 1988), which suggests multiple potential dispersers in the Messel ecosystem for this particular seed. The fact that two specimens of the zygodactylid *Primozygodactylus major* ingested the same type of seeds (Vitaceae) may indicate a feeding preference of this bird species for grapes. The Zygodactylidae are comparatively common among the small arboreal birds from Messel. These birds have a characteristic foot morphology, and unlike in passerines, their sister taxon, the fourth toe was permanently reversed. Together with the long legs, this grasping foot was well suited for foraging in lianas and climbing plants, which constitute the predominant growth forms of extant Vitaceae. Seeds of multiple species of the Vitaceae are known from the early Eocene British London Clay (Chandler 1961; Collinson 1983), where zygodactylids are also among the more common small arboreal birds (Mayr and Kitchener 2023).

The occurrence of seeds in the stomach contents of three individuals of stem group rollers (Coracii, Coraciiformes) from Messel is notable, because the extant species of this clade are predominantly carnivorous, feeding on insects and vertebrates, even though European rollers “commonly consume grapes in the Ukraine and figs in the Mediterranean” (Fry 2001: 359). The taxa *Primobucco* and *Eocoracias* are successively closer to crown group Coracii (Mayr et al. 2004; Clarke et al. 2009), so that an at least partially frugivorous diet may have been plesiomorphic for the Coracii.

On average, Cretaceous angiosperms had much smaller seeds than early Cenozoic ones (Tiffney 1984; Eriksson et al. 2000; Friis et al. 2011), and it has been debated whether this increase of angiosperm seed size was due to the early Cenozoic radiation of mammalian and avian dispersers (disperser or coevolution hypothesis; Tiffney 1984), or whether it was due to changes in environmental conditions and vegetation structure, which in turn favored animal-mediated distribution (recruitment hypothesis; Eriksson et al. 2000). The scarcity of early Cenozoic vertebrate fossils with ingested seeds has long impeded a direct assessment of these hypotheses.

Even though it was hypothesized that dispersal by birds might have contributed to the size increase of angiosperm seeds towards the early Cenozoic (Tiffney 1984; Eriksson 2008), the Messel fossils do not support a coevolution hypothesis. Most seeds ingested by the birds are small to very small, and in the size ranges of Mesozoic angiosperm seeds (see data in Tiffney 1984). Actually, the size of most seeds associated with the birds from Messel is not larger than those found in the stomach content of the Early Cretaceous *Jeholornis*, where ingested seeds measure 8–10 mm (Zhou and Zhang 2002). Moreover, the Messel birds were gulpers that swallowed whole disseminules, which are likely to have passed the gut undamaged; a larger seed size would, therefore, not have had positive selective effects on the survival chances of the plant embryo. Currently, no large arboreal avian frugivores are known from the early Cenozoic (Mayr 2017a), so that the size increase of angiosperm seeds towards the early Cenozoic is unlikely to have been correlated with a dispersal by birds.

The presence of seeds of the Vitaceae in multiple stomach contents of taxa of the Telluraves is notable and indicates that grapes played a major role in the diet of some of these birds. Seeds of the Vitaceae have a distinctive morphology (Chen and Manchester 2011), and their oldest fossils, which also constitute the earliest record of the family as such, stem from the Late Cretaceous of India (Manchester et al. 2013). The radiation of the Telluraves, by contrast, took place in the early Cenozoic (Mayr 2017b: 93; Field et al. 2018; Stiller et al. 2024), so that the origin of the Vitaceae and their characteristic seeds predates that of the avian taxa from Messel, which fed on grapes. This provides further evidence for the recruitment hypothesis and suggests that at least early Eocene telluravians exploited an existing diversity of edible fruits.

The data from Messel do not exclude the possibility that mutualistic adaptations between frugivorous birds and ornithochorous plants already occurred earlier in the Cenozoic. Earlier diverging lineages of Neoaves, such as the Gruiformes – to which the Messelornithidae belong –, probably go back to the Late Cretaceous (Stiller et al. 2024), and Mesozoic stem lineage representatives of these birds may have been opportunistic feeders of angiosperm fruits. However, if a coevolution with vertebrate dispersers contributed to the size increase of angiosperm seeds, frugivorous early Cenozoic mammals (Collinson and Hooker 1991) may have played a more important role.

Current evidence indicates that there were only few – if any – frugivorous birds in Early Cretaceous ecosystems, and that these fed on gymnosperm plants. Contrary to previous inferences (Benton et al. 2022), birds are therefore unlikely to have contributed to the Cretaceous Terrestrial Revolution, and other vertebrates were probably more important as seed dispersers in Mesozoic ecosystems.

The Messel fossils open a unique window into the diversity of early Cenozoic frugivorous birds and the angiosperm plants to whose dispersal they contributed. However, there exists a large temporal gap of more than 70 million years between the ecosystems from Messel and the Chinese Jehol Biota. As yet, no other early Cenozoic and Cretaceous fossil localities yielded birds with stomach contents containing seeds and it is to be hoped that future fossils will shed more light on the earliest stages of frugivory in neornithine birds. 

## Data Availability

No datasets were generated or analysed during the current study.
